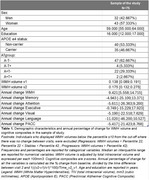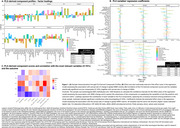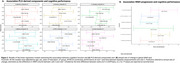# Distinct risk factors drive white matter hyperintensity progression which relates to cognitive performance in individuals at risk of Alzheimer's disease

**DOI:** 10.1002/alz70856_106766

**Published:** 2026-01-11

**Authors:** Patricia Genius, Blanca Rodríguez‐Fernández, Mahnaz Shekari, Gonzalo Sánchez‐Benavides, Gwendlyn Kollmorgen, Clara Quijano Rubio, Carole H Sudre, Marta Cirach, Mark Nieuwenhuijsen, Marc Suárez‐Calvet, Arcadi Navarro, Juan Domingo Gispert, Natalia Vilor‐Tejedor

**Affiliations:** ^1^ Barcelonaβeta Brain Research Center (BBRC), Pasqual Maragall Foundation, Barcelona, Spain; ^2^ Centre for Genomic Regulation (CRG), Barcelona Institute of Science and Technology (BIST), Barcelona, Spain; ^3^ PhD programme in Bioinformatics. Doctoral School. University of Vic ‐ Central University of Catalonia (UVic‐UCC), Vic, Spain; ^4^ Hospital del Mar Research Institute, Barcelona, Spain; ^5^ Centro de Investigación Biomédica en Red de Fragilidad y Envejecimiento Saludable (CIBERFES), Instituto de Salud Carlos III, Madrid, Spain; ^6^ Roche Diagnostics GmbH, Penzberg, Germany; ^7^ Roche Diagnostics International Ltd, Rotkreuz, Zug, Switzerland; ^8^ School of Biomedical Engineering and Imaging Sciences, King's College London, London, United Kingdom; ^9^ Department of Neurodegenerative Disease, The Dementia Research Centre, UCL Queen Square Institute of Neurology, London, United Kingdom; ^10^ Hawkes Institute, University College London, London, United Kingdom; ^11^ Unit for Lifelong Health and Ageing, Department of Population Science and Experimental Medicine, University College London, London, United Kingdom; ^12^ Centro de Investigación Biomédica en Red de Epidemiología y Salud Pública (CIBERESP), Madrid, Spain; ^13^ Neurochemistry Laboratory, Department of Clinical Chemistry, Amsterdam Neuroscience, Vrije Universiteit Amsterdam, Amsterdam UMC, Amsterdam, Netherlands; ^14^ ISGlobal, Barcelona Institute for Global Health ‐ Campus MAR, Barcelona Biomedical Research Park, Barcelona, Spain; ^15^ Hospital del Mar Research Institute, Barcelona, Barcelona, Spain; ^16^ BarcelonaBeta Brain Research Center (BBRC), Barcelona, Spain; ^17^ Institute of Evolutionary Biology (CSIC‐UPF), Department of Experimental and Health Sciences, Universitat Pompeu Fabra, 08003, Barcelona, Spain; ^18^ Institució Catalana de Recerca i Estudis Avançats (ICREA), Barcelona, Spain; ^19^ AstraZeneca, Barcelona, Spain; ^20^ Department of Genetics, Radboud Medical University Center, Nijmegen, Netherlands

## Abstract

**Background:**

White matter hyperintensities (WMHs) are a hallmark of cerebral small vessel disease (CSVD) linked to cognitive impairment and risk of dementia. Several risk factors are associated with WMH, but their contribution to WMH progression remains unclear. This study aimed to identify risk factors driving WMHs changes in middle‐aged individuals and to examine their impact on cognitive decline.

**Method:**

Global WMHs and cognitive composites were evaluated at baseline and after three years on 75 cognitively unimpaired individuals at risk of Alzheimer's disease (AD) whose WMH volumes increased (Progressors: ΔWMHv2‐v1 > 10+Percentile ΔWMHv2‐v1=0). CSF biomarkers, exposure to air pollutants, AD pathway‐specific polygenic risk scores (PRS) and cardiovascular risk factors were evaluated as predictors of the annual rate of change of global WMH, which was assessed as a predictor of cognitive performance. Partial least squares regression identified latent factors describing predictor‐outcome covariance. Latent factors and WMH change were used in independent linear regression models to predict cognitive performance.

**Result:**

Global WMHs increased by 9.5%, while cognitive composites declined annually by 1–11% [Table 1]. Five latent factors explained 56% of the variance in risk factors, though their stability was limited, with only a few significant loadings after bootstrapping (CI 95%) [Figure 1A]. Key predictors of WMH progression included higher genetic risks for WMH and AD (via the stimulus signaling pathway) and notably lower educational attainment, even when paired with low levels of other risk factors [Figure 1B]. Component 3, linked to baseline risk factors (e.g. Centiloid, CSF Aβ42/40)[Figure 1C], predicted lower language and PACC scores after 3 years [Figure 2A]. While WMH change did not mediate this association, it correlated with worse PACC (*p* = 0.057) and memory performance (ꞵ= ‐0.1 [‐0.188, ‐0.011], *p* = 0.028) [Figure 2B].

**Conclusion:**

WMHs progression is linked to subclinical cognitive decline in cognitively unimpaired individuals, for which low educational attainment and risk of AD via signaling pathways drive WMH progression. Remarkably, when combined with other risk factors, these variables predict worse PACC outcomes. These findings highlight the need to work with larger sample sizes to identify covariance patterns in modifiable and non‐modifiable risk factors related to cerebrovascular damage and cognitive decline.